# Acute Haematogenous Revision Total Knee Arthroplasty Infection by Streptococcus canis Treated by Debridement, Antibiotics, and Implant Retention: A Case Report

**DOI:** 10.7759/cureus.58247

**Published:** 2024-04-14

**Authors:** Gloria Pedemonte-Parramón, Esteban Reynaga, Sònia Molinos, Vicente López-Pérez, José A Hernández-Hermoso

**Affiliations:** 1 Department of Orthopaedic Surgery and Traumatology, Hospital Universitari Germans Trias i Pujol, Badalona, ESP; 2 Department of Infectious Diseases, Hospital Universitari Germans Trias i Pujol, Badalona, ESP; 3 Department of Microbiology, Hospital Universitari Germans Trias i Pujol, Badalona, ESP; 4 Department of Surgery, Faculty of Medicine, Universitat Autònoma de Barcelona, Bellaterra, ESP

**Keywords:** ceftriaxone, implant retention, debridement, c-reactive protein, streptococcus canis, haematogenous infection, total knee arthroplasty, prosthetic joint infection

## Abstract

Prosthetic joint infections (PJIs) are one of the most feared complications by orthopaedic surgeons. Haematogenous PJI represents an important part of PJI cases. *Streptococcus canis* is an extremely rare cause of haematogenous PJI and its medical and surgical treatment and prognosis are not well established.

We present a 79-year-old female patient who had a revision total knee arthroplasty (rTKA) surgery three years before. She was admitted to the hospital referring to three days of knee pain, restricted range of motion, and fever. Blood tests demonstrated leukocyte and C-reactive protein elevation. Joint fluid aspiration showed elevated white blood cell count with a high neutrophil differential and its conventional culture was positive for *Streptococcus canis*. She did not have pets but she took care of her daughter's dog. An acute haematogenous infection of the rTKA was diagnosed and treated with debridement, antibiotics (eight weeks of IV ceftriaxone), and implant retention (DAIR). After one year, the patient remains clinically asymptomatic without changes on X-rays and with normal serum levels of inflammatory blood markers.

*Streptococcus canis* has to be kept in mind as a possible cause of haematogenous TKA infection in patients who have contact with domestic pets and patients should be asked for this contact. We recommend DAIR as a viable treatment option for this type of infection, which may show excellent results.

## Introduction

Total knee arthroplasty (TKA) is one of the most performed orthopaedic surgical procedures [1]. Nonetheless, it is not free of complications, being one of the most feared prosthetic joint infections (PJIs) [2,3], which is associated with high morbidity [1,4] and a non-negligible mortality [1,4].

The incidence of primary prosthetic knee infections ranges between 1% and 2% but increases up to 5-6% in revision surgeries [5]. Prosthetic infections are typically classified according to the Tsukayama classification into four types: immediate post-surgery, chronic, haematogenous, and positive intraoperative cultures [6]. The haematogenous PJI type represents up to 20-35% [7]. The most common causative microorganisms of haematogenous TKA infections are *Staphylococcus aureus* and *Streptococcus spp* [7,8].

PJI due to commensal microorganisms in animals, such as *Streptococcus canis*, are rare. There are only two well-described cases of *Streptococcus canis* PJI in the literature [9]. The first one was a patient with a haematogenous TKA infection associated with systemic illness, who was treated with two-stage replacement surgery and six weeks of intravenous vancomycin [10]. The second was a patient with a postoperative acute hip PJI, who was treated by one-stage revision surgery, followed by six weeks of IV cefazolin [4]. Both cases were ultimately linked to close regular contact with their pet dog. No *Streptococcus* *canis* PJI of haematogenous origin treated by debridement, antibiotics, and implant retention (DAIR) has been reported in the literature.

Depending on the time of onset and the status of implant fixation, the recommended surgical treatments in PJI of haematogenous spread are DAIR and one- or two-stage revision surgery [3,11,12]. DAIR has a non-negligible risk of failure, depending on the causative microorganism [11,12]. *Staphylococcus aureus* is a microorganism with a higher risk of failure [11-13]. Nevertheless, *Streptococcus spp *are also associated with worse results with DAIR, with up to 50% of failures [11,12]. However, the presence of *Streptococcus canis* in these series is practically null, so it is unknown if it will follow the same behaviour.

Our objective is to describe the clinical presentation, the approach, and the treatment and result of an unusual clinical case of a *Streptococcus canis* haematogenous infection of a revision TKA treated with DAIR.

## Case presentation

**Figure 1:**
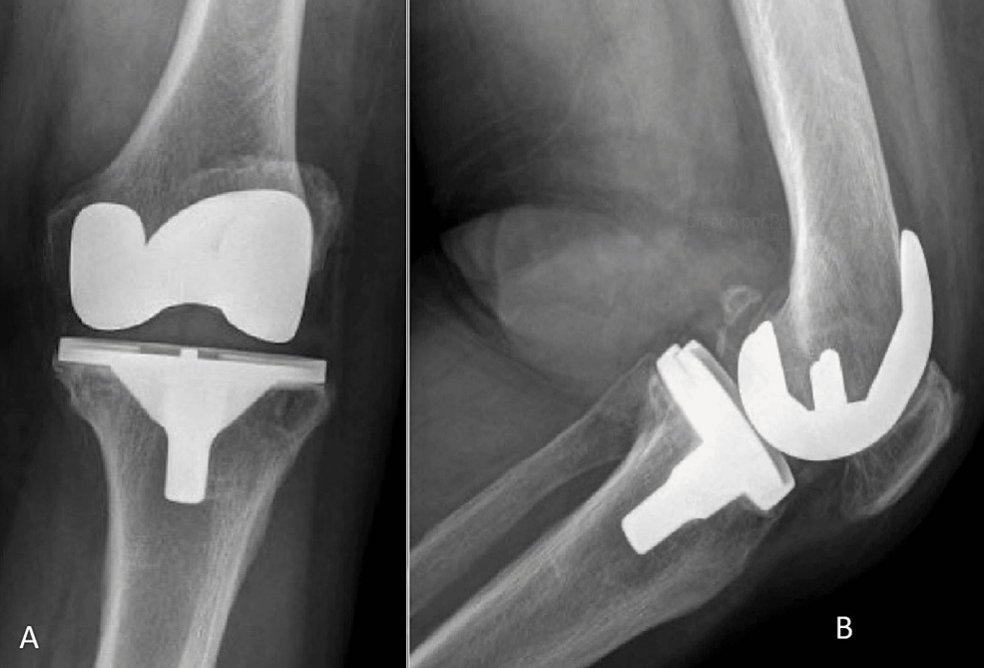
Anteroposterior (A) and lateral (B) radiographs of the primary total knee arthroplasty.

**Figure 2:**
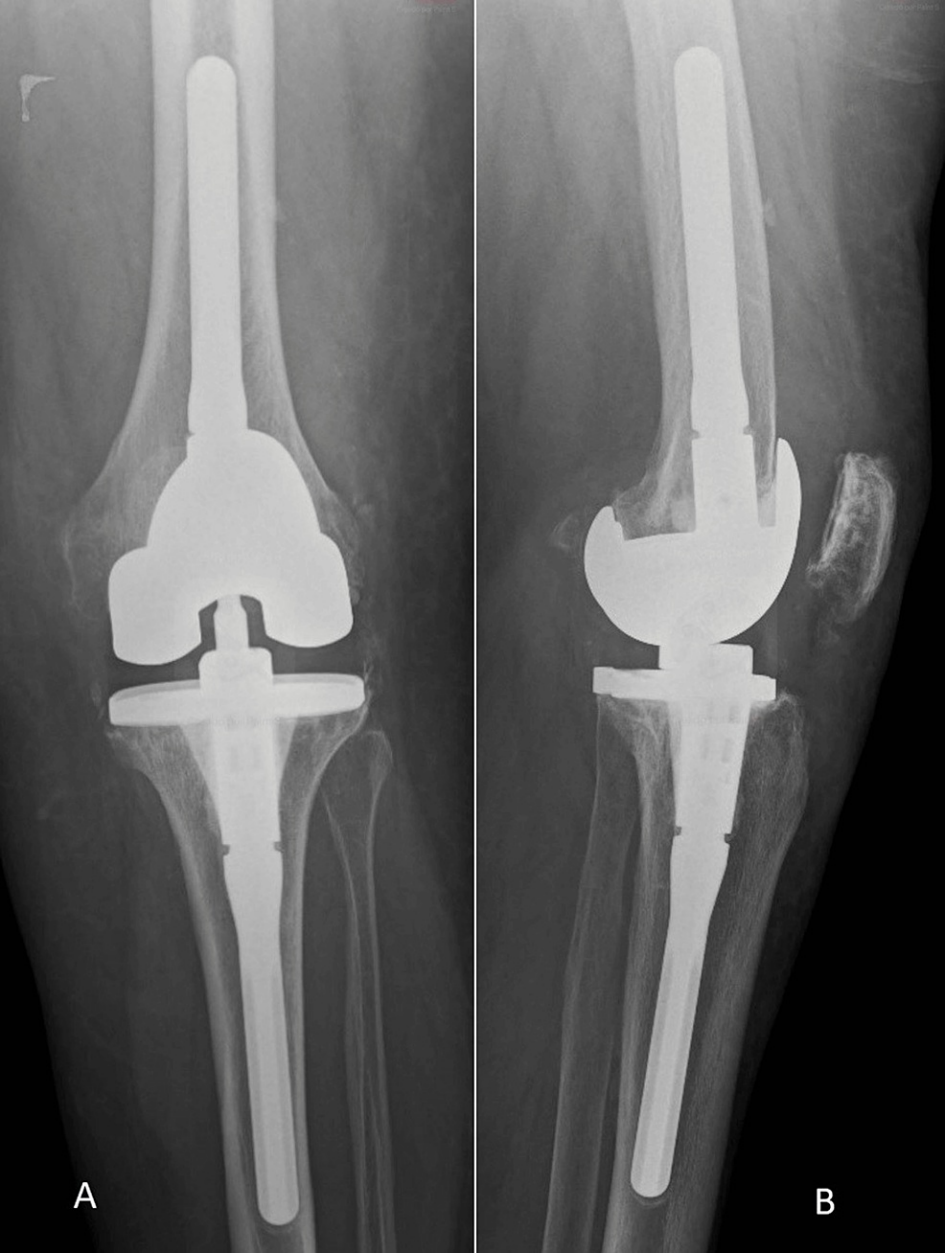
Anteroposterior (A) and lateral (B) radiographs of the revision total knee arthroplasty previous to the haematogenous infection.

**Figure 3:**
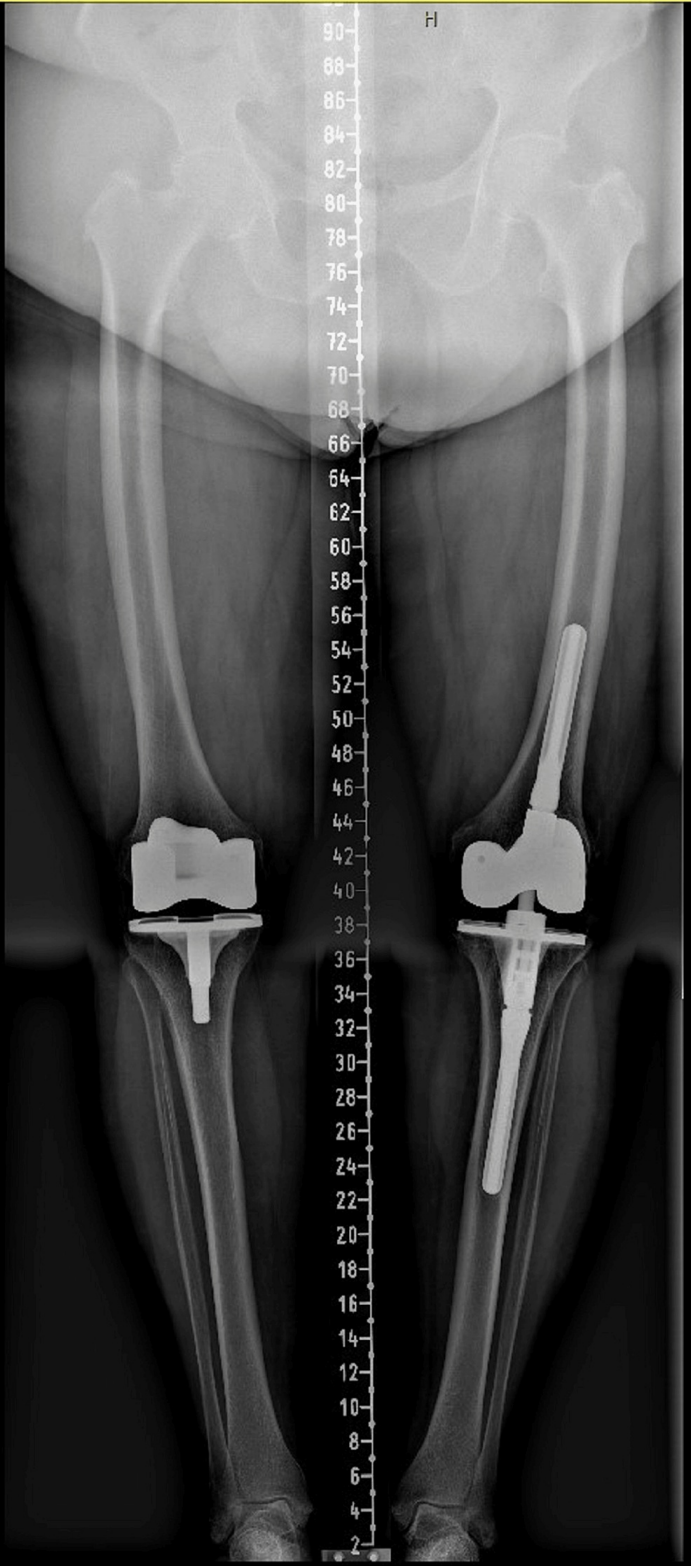
Full weight-bearing lower leg radiograph of the revision total knee arthroplasty previous to the haematogenous infection.

A 79-year-old Spanish female, with a medical history of hypertension, atrial fibrillation, chronic bronchitis, obstructive sleep apnoea, and obesity class III [14], on chronic treatment with enalapril 5 mg/24 hours, amiodarone 200 mg/24 hours, apixaban 5 mg/12 hours, and ipratropium bromide 20 mcg two inhales/24 hours, went to the traumatology emergency department. She had a left total knee replacement with a Multigen Plus® CR (LimaCorporate®, Udine, Italy) for knee osteoarthritis in 2009 (Figure 1). She received a one-stage revision surgery with an antibiotic (2 g gentamicin) cemented NexGen® Legacy® Constrained Condylar Knee (Zimmer Biomet®, Zug, Switzerland) with femoral and tibia stems in 2020 for aseptic loosening of the TKA (Figures 2, 3). Intraoperative cultures and histology were negative for infection at that time. The patient gave written consent for personal data concerning the case to be submitted for publication.

She reported continuous left knee pain, not well localized, chills, and limited knee range of motion (ROM) that began three days ago, and since then being unable to walk. She denied previous knee pain or inflammatory signs, and no fever or other associated symptoms were described. She denied recent urine, respiratory, or skin infections or wounds. On physical exam, she was haemodynamically stable with a fever up to 38°C. Warmth, redness, and swelling of the left knee were appreciated and a limited ROM of 0-30° was noted. No radiolucent lines surrounding the prosthesis were evident and a well-alignment implant without changes in the position over time was appreciated on X-ray. Blood cell count revealed an elevated leukocyte count of 14,800 x109L and C-reactive protein (CRP) of 319.8 mg/L.

Blood and joint fluid aspiration cultures of the knee under aseptic conditions were ordered due to high suspicion of PJI. Joint fluid showed cell counts of 81,250 cells/mm3 with a 94% neutrophil differential, a glucose level of 10 mg/dL, a protein level of 47 g/L, and a lactate dehydrogenase of 7140 U/L. The diagnosis of PJI was done in light of the clinical evaluation and laboratory investigations, which met the International Consensus on Orthopedic Infections criteria for the diagnosis [15]. At 24 hours, the conventional joint fluid cultures sent in an aerobic and anaerobic blood culture bottle (BD BACTEC™, Becton, Dickinson and Company, Franklin Lakes, NJ) isolated a *Streptococcus canis* microorganism. Blood cultures were negative. Due to the microorganism being isolated, the patient was asked for domestic animals. She did not have pets but she took care of her daughter's dog often, and the dog repeatedly bit or licked her extremities. However, no skin bites or scratches were evident during the physical exam at hospital admission.

Surgical treatment

Immediately after the diagnosis, we calculated the risk of DAIR failure, which in this particular patient was low-moderate, getting 2 points in the CRIME80 score [12]. The DAIR procedure was done under general anaesthesia and ischaemia. The tourniquet was inflated after two to three minutes of elevation of the lower leg. An iterative anterior knee approach was performed following the previous scar. Joint fluid aspiration was done prior to the medial arthrotomy and was sent for culture, cell count, and biochemistry test. Polyethylene exchange, complete synovectomy, extensive debridement, including the posterior compartment and irrigation with one litre of dilute povidone iodate 3.5% and nine litres of sterile normal saline solution, were carried out. The superficial drape layer of the surgical field was exchanged and a new polyethylene NexGen® Legacy® Constrained Condylar Knee (Zimmer Biomet®, Zug, Switzerland) of the same size was implanted (14 mm), achieving correct stability. Seven intraoperative cultures samples were taken from the more affected parts: the synovia in the suprapatellar pouch, the medial and lateral gutters, the intercondylar notch, the Hoffa fat pad, the posterior capsule, and the last one was the sonication of the polyethylene. The incision was closed in a layered fashion using #2 Vicryl® (90% glycolide and 10% L-lactide) sutures for deep layers, # 2/0 for the subcutaneous layer, and staples for the skin. One intra-articular drain was used.

Antibiotic treatment

After the culture samples were obtained, empiric antibiotic treatment with IV ceftazidime 2 g/eight hours and vancomycin 1 g/12 hours was started using a peripherally insertable central catheter inserted previously. The intraoperative cultures confirmed the suspected diagnosis 72 hours after the surgery, isolating *Streptococcus canis* in seven of the eight cultures. The antibiotic treatment was changed to ceftriaxone IV 2 g/24 hours. Due to the good clinical evolution, 14 days after the surgery, the IV antibiotic was discontinued and switched to oral levofloxacin 750 mg/24 hours and rifampicin 600 mg/24 hours. After the change to oral antibiotics, the patient presented diarrhoea. *Clostridium difficile *toxins and stool culture were negative, so antibiotic intolerance was diagnosed and oral antibiotics were discontinued and switched again to IV ceftriaxone 2 g/24 hours for six weeks.

Complications

Seven days after the hospital admission, the patient presented an episode of acute dyspnoea and blood oxygen desaturation up to 90%. The chest X-ray showed a bilateral pleural effusion and interstitial oedema. Acute heart failure was the diagnosis, which resolved after oxygen therapy and depletive treatment with furosemide. Sixteen days after the hospital admission, due to diarrhoea secondary to antibiotic intolerance, a second heart failure decompensation was diagnosed, which was resolved with the same medical treatment.

After 18 days of the hospital admission, the patient was discharged at home with a home hospitalization service to control the intravenous antibiotic treatment and the medical condition.

Follow-up

**Figure 4:**
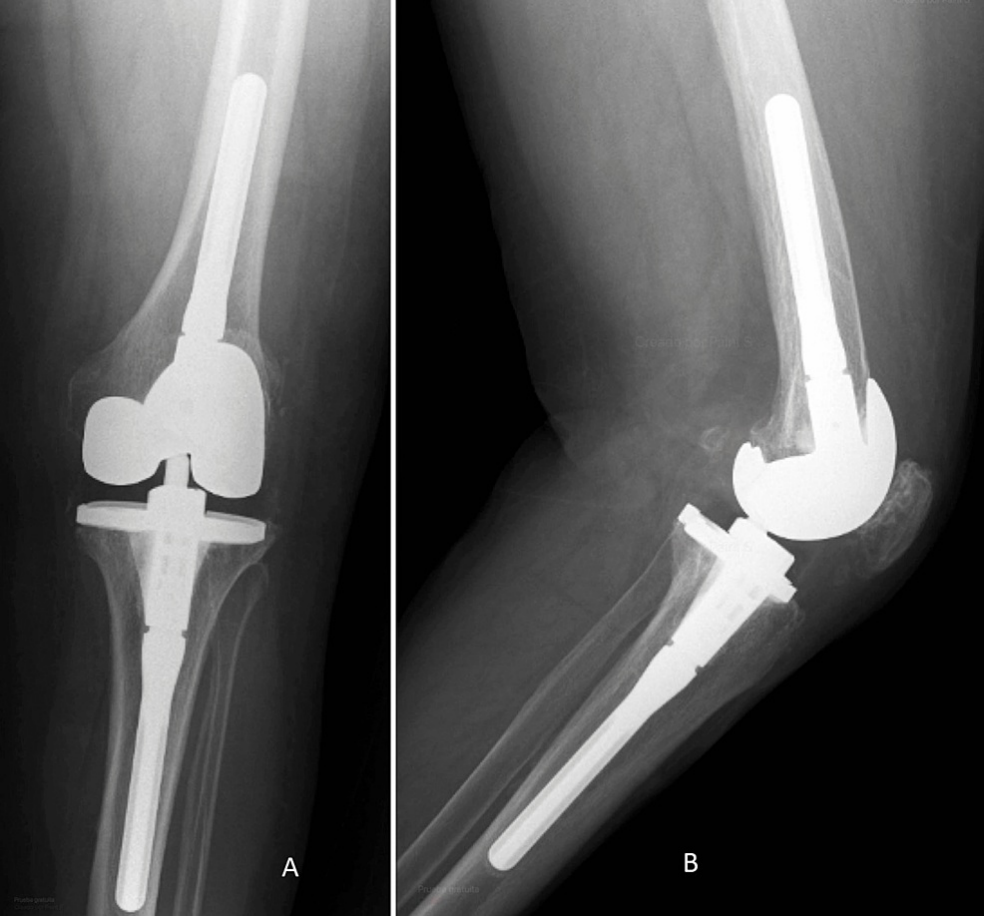
Anteroposterior (A) and lateral (B) radiographs of the revision TKA after the DAIR procedure at one year of follow-up. TKA: total knee arthroplasty; DAIR: debridement, antibiotics, and implant retention.

One year after the DAIR procedure, the patient remains clinically asymptomatic. She is able to walk an unlimited distance without technical assistance. She can walk up and down stairs with the handrail. Her knee does not present any clinical sign of inflammation and has a ROM of 0-100º, without varus-valgus or anteroposterior instability. No radiolucency lines or signs of implant mobilization are appreciated on the anteroposterior and lateral knee X-rays (Figure 4). The blood test showed normal values of leukocyte cell count (6,500x109L) and serologic inflammatory biomarkers (CRP of 4.40 mg/L and erythrocyte sedimentation rate (ESR) of 20 mm/h) at one year postoperatively.

## Discussion

We present a case of an acute haematogenous TKA infection due to an uncommon causative microorganism in humans, namely, *Streptococcus canis*. This microorganism can cause infections in humans who usually have had contact with domestic pets [4,9,10]. This is the first reported clinical case of an acute haematogenous revision TKA infection treated with the DAIR procedure achieving an excellent result.

PJIs are one of the most feared and serious complications for orthopaedic surgeons [2,3] due to the associated morbidities [1,4] as well as the non-negligible risk of mortality [1,4], their incidence being up to 5-6% in revision TKA [5]. Tsukayama et al. [6] classified PJI into four types: immediate post-surgery, chronic, haematogenous, and positive intraoperative cultures. The incidence of PJI of haematogenous origin accounts for 20-35% of the PJIs [7]. The most frequent places of origin of the microorganisms that spread through the vascular stream are the cardiovascular system, the skin and soft tissue, the oral cavity, and the gastrointestinal or urogenital tracts [7,8]. *Staphylococcus aureus* and *Streptococcus spp* are the most common isolated microorganisms [7,8]. Acute haematogenous PJI due to animal commensal microorganisms, such as *Streptococcus canis*, is very rare [9,16], being <1% of streptococcal PJI [4]. There are only two cases reported in the literature [9].

*Streptococcus canis* is a G beta-hemolytic species of *Streptococcus* first isolated in dogs. It is a commensal microorganism of the skin and mucous membranes of the respiratory tract, the rectum, and the genital tract of cats and dogs [9]. It can cause opportunistic infections in animals like skin infections and other severe clinical diseases such as meningitis or myocarditis [9,16,17]. It can rarely cause infections in other mammals and very rarely in humans [9,16]. The pathophysiology of the infection in humans is still unknown [9]. Despite that, it is believed to be through the faecal contamination of the environment or by direct inoculation from a pet to humans through the contamination of wounds or bites or skin scratches [9,16,17]. This direct transmission makes *Streptococcus canis* to be considered as a zoonotic pathogen [9,16].

Other reported cases of *Streptococcus canis* infections, as in our case, did not have a clear portal of entry for the microorganism [4,9,10,18,19]. The possibility of *Streptococcus canis* colonization in pet owners is unclear [4,9], and it has not been demonstrated as a risk factor for PJI [4]. It has to be asked for and this possibility needs to be taken into account in pet owners because, as in our case, contact with pet dogs was present in all reported cases [4,10]. Other risk factors have been described such as aging or immunosuppression [9,19]. *Streptococcus canis* infections in humans may be associated with soft tissue ones such as cellulitis or abscesses, and in atypical cases, they can cause bacteremia and septic emboli, pneumonia, or endocarditis [9].

Clinically, *Streptococcus canis* PJI infection can present as an acute infection post surgery [4] or it can be haematogenous [10], with a fever over 38°C and with the patient feeling unwell and associated with a systemic illness that can cause severe septicemia with hypotension [10]. Local signs of inflammation, such as warmth, erythema, swelling, and pain are usually present [4,10]. Serological blood markers of infection (leukocytes, CRP, and ESR) are normally elevated and the analyses of the joint fluid aspiration usually reveal an elevation of nucleated cell count with a high percentage of neutrophils. So, the clinical presentation is in agreement with the one described for acute PJI [15].

*Streptococcus canis* can be identified in conventional cultures of the preoperative synovial joint fluid aspiration, as in our patient, or the intraoperative tissue samples [4]. Nevertheless, it can be difficult to identify with conventional cultures [10] or it can be confused with *Streptococcus dysgalactiae* [9,16,20], which makes the real incidence of *Streptococcus canis* in humans unknown. For this reason, it is recommended to use new diagnostic techniques such as polymerase chain reaction or next-generation sequencing [10], which are promising methods to facilitate its identification [9,10,16].

Acute haematogenous PJI can be treated by different surgical techniques depending on the time of evolution, the type of microorganism, and the presence of a well-fixed or loose implant. In acute PJI, DAIR is a well-accepted treatment and probably the most desired by surgeons since it tries to maintain a functional and stable implant [3,11,12]. Although DAIR has shown good results in streptococcal prosthetic joint infections [11,12], it has never been reported in *Streptococcus canis* acute PJI, since the previous infections described were treated with one- [4] or two-stage [10] revision surgery. However, when a difficult-to-treat microorganism is isolated, when a fistula is observed, or when there is an implant failure, the recommended treatment is revision surgery, which can be done as a one- or two-stage procedure [3,11].

DAIR failure risk is not negligible in acute infections and increases in haematogenous ones by up to 50% [3,11,12]. To calculate preoperatively the chances of DAIR failure, different scores have been developed and are under discussion [11], such as the KLIC (kidney, liver, index surgery, cemented prosthesis, and C-reactive protein value) score for acute PJI [13] and the CRIME80 score for haematogenous PJI [12]. Although no other *Streptococcus canis* acute PJI cases reported in the literature were treated with DAIR, we decided to perform it because the implant was well fixed and the risk of failure in our patient was low, following the CRIME80 score [12]. It is advisable to know the causative microorganism and their susceptibility to antibiotic treatment to ensure the success of DAIR and any other surgical treatment [11], but this is not always possible before the surgery due to the emergency of the treatment. *Staphylococcus aureus* is the one with a higher risk of failure [11-13]. However, *Streptococcus* *spp* also can have a high risk of DAIR failure, i.e., up to 50% depending on the cases [11,12]. The presence of *Streptococcus canis* in these series is practically null, so it cannot be assured that it will follow the same behaviour.

*Streptococcus canis* is sensitive to common antibiotics such as penicillin or cephalosporins, clavulanic acid, or vancomycin with which it can be treated [4,9,20]. It is still unclear which is the best route and duration of antibiotic treatment, but recent guidelines [11] suggest good results in PJI with a six- to eight-week course of antibiotics, being intravenously the first two weeks followed by four to six weeks of oral antibiotic. Further, in DAIR, the use of antibiotics against biofilms, such as rifampicin in staphylococcal PJI or fluoroquinolones in gram-negative PJI, is important [11]. Occasional resistance to gentamicin and rifampicin has also been reported in *Streptococcus canis* [9]. Due to its rarity, very little is known about the best antibiotic treatment for *Streptococcus canis* PJI. Good results have been reported using IV vancomycin [10] and cefazolin [4] associated with one- [4] or two-stage [10] revision surgery in *Streptococcus canis* PJI. For the first time, we report an excellent result of a revision TKA acute haematogenous infection treated with DAIR and IV ceftriaxone for eight weeks.

## Conclusions

*Streptococcus canis* has to be kept in mind as a possible cause of haematogenous TKA infection in patients who have contact with domestic pets and patients should be asked for this contact. The DAIR procedure can achieve excellent clinical results without infection relapse in the treatment of *Streptococcus canis* acute haematogenous TKA infection. As with any PJI, the treatment must be individualized taking into account patient risk factors and comorbidities, skin, and implant fixation status.
